# Supplementary material for: Securing Data in Multimode Fibers by Exploiting Mode-Dependent Light Propagation Effects
Source: Research (Wash D C). 2023 Feb 27;6:0065. doi: 10.34133/research.0065 (PMC10013962; doi:10.34133/research.0065)
Supplement: Supplementary Materials — Fig. S1. Investigation on SVD-based channel diagonalization in dependence of varying MMF lengths. Fig. S2. Optical setups used for our experiments. Fig. S3. Considered wiretap channel model with a passive eavesdropper. [file research.0065.f1.pdf]

# Supplementary Material

## 1 Characterisation of channel diagonalisation through SVD on varying fibre lengths

Diagonalisation of the TM allows Alice to transmit data to Bob specificially on certain spatial data streams. By multiplying the prescrambling matrix $U^H$ to her confidential message, Alice obtains the required mode combination, which she should launch with the SLM. Contrary to Eve, Bob does not have to perform channel inversion, but can decipher the received message by simply multiplying it with his retrieval matrix $V$. This procedure compensates for modal crosstalk, the quality of which depends in particular on the diagonalisability. The question is: how well can Alice and Bob diagonalise their TM, i.e. their channel? For this investigation, MMFs of different lengths are chosen as data links between Alice and Bob, which are diagonalised by SVD. An example of a diagonalised TM is shown in Fig. S1a. A high level of diagonalisability is achieved when a main diagonal entry of the TM corresponding to a data stream has a high value relative to its average background. For this purpose, the TM diagonalised by SVD is investigated row by row and the SNR for the respective data stream is calculated:

$$\begin{aligned} \mathrm{SNR} &= 10 \cdot \log_{10}\left(\frac{P_{\mathrm{data}}}{P_{\mathrm{background}}}\right) \mathrm{dB} \\ &= 10 \cdot \log_{10}\left(\frac{\rho^2_{\mathrm{data}}}{\mu^2_{\mathrm{background}}}\right) \mathrm{dB}. \end{aligned} \tag{1}$$

The SNR is defined as relation between the power of the data stream $P_{\mathrm{stream}}$ and the background power $P_{\mathrm{background}}$. The power in the data stream is determined by the square of the main diagonal element $\rho_{\mathrm{data}}$. The power in the background is determined by the average of the squared background elements $\mu_{\mathrm{background}}$. The results are shown in Fig. S1b. They are based on 5 consecutive measurements for different MMF lengths (1 m, 10 m, 100 m).

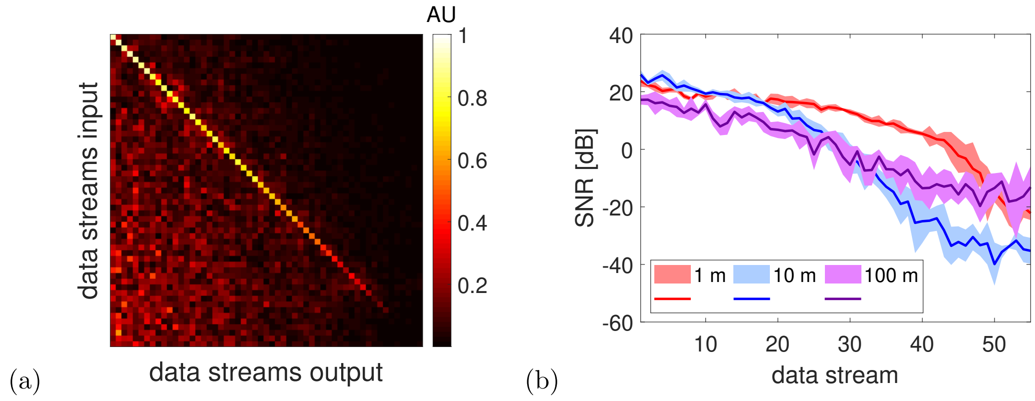


Figure S1: Investigation on SVD-based channel diagonalisation in dependence of varying MMF lengths. The diagonalisation is performed as shown in Fig. 3. (a) Measured TM between Alice and Bob at 1 m MMF after diagonalisation. (b) Evaluation of the SNR over available data streams. Mean values are indicated by the respective curve, whereas the standard deviation is given by the filled area around the curve. For each MMF length, 5 measurements were performed.

It can be shown for all fibre lengths investigated that channel diagonalisation using SVD can generate data streams with high SNR of up to 26 dB. This result corresponds to other values from literature regarding channel estimations of SDM systems that operate with a single MMF core over 45 transverse modes [1]. However, the number of available data streams with high SNR varies due to MDL. While 44 data streams with positive SNR are available at 1 m MMF, there are only 24 at 100 m. We attribute this to the step-index profile of the MMFs under test. Step-index fibres are known to provide less stable

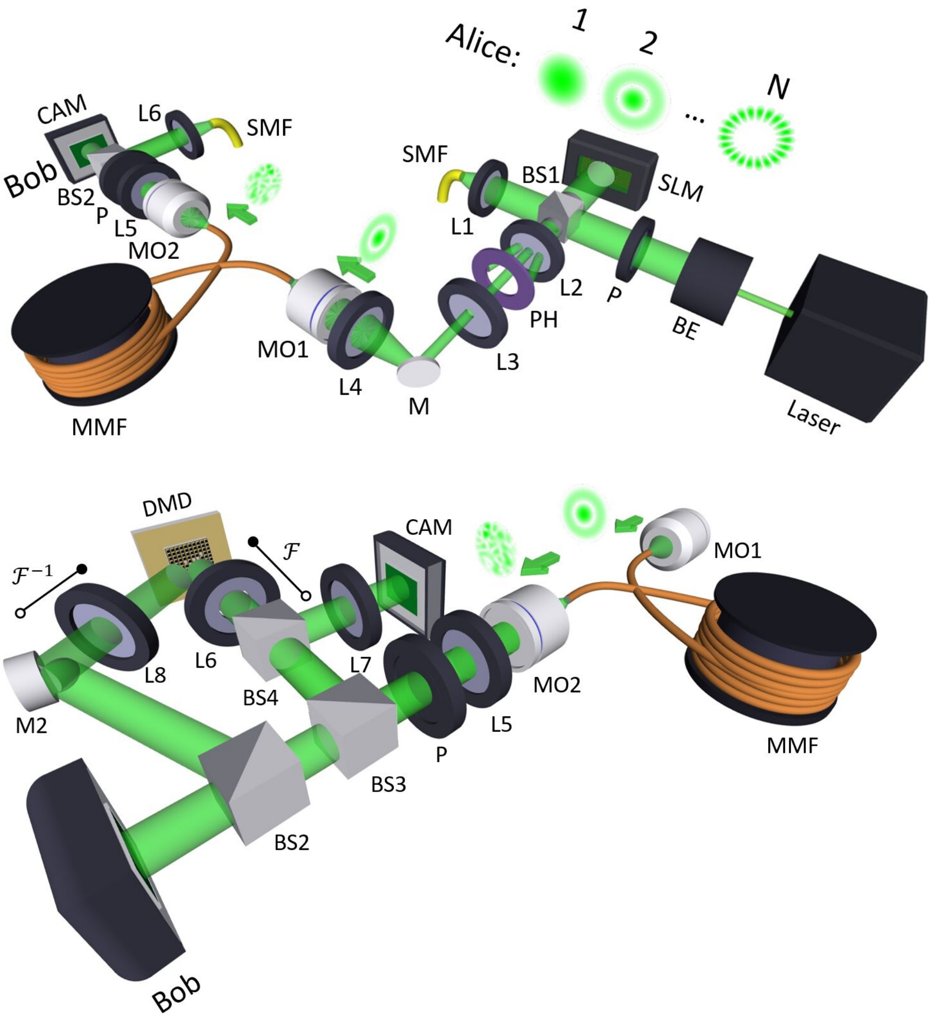


(a)

(b)

Figure S2: Optical setups used for our experiments. (a) Scheme for TM measurements with external reference. This setup was used for experiments on up to 10 m MMF. For measurements including Eve and a tapped MMF, the receiver-side was simply copied for Eve's access. BE: beam expansion; P: linear polarisation filter; BS: beam splitter cube; L: lens; PH: pinhole; M: mirror; MO: microscope objective; CAM: CMOS camera. (b) Common-path extension for the receiver-side for MMFs with increased length. As temporal fluctuations increase significantly in their frequency with longer fiber links, stability of the measurement scheme becomes an issue. Here, we propose a common-path solution applied to Bob's side with which we have conducted diagonalisation experiments with 100 m MMF. DMD: digital micromirror device.

light transport and are more susceptible to external influences such as temperature and mechanical stress compared to GRIN. With GRIN, the refractive index profile is optimised for the transmission of multiple transverse modes. Thus, we expect that more data streams are suitable for data transmission at long GRIN MMFs.

## 2 Optical Setup

In Fig. S2a, the optical setup we used for our experiments is shown. A laser beam (LaserQuantum,

Torus, 532 nm) is expanded that it exceeds the SLM (Holoeye, PLUTO) display and a plane illumination can be assumed. We use only one linear polarisation state for TM measurement, which means that we use only 50 % of the available power. However, the system can be straightforwardly extended to consider the orthogonal state, as well [2], [3]. Before the SLM is illuminated, the beam is split into object and reference path by beam splitter BS1. The reference is coupled into an SMF via lens L1. The length of the SMF is adjusted so that object and reference beam are coherent with each other and a sufficient fringe contrast is provided. Superpixel phase masks are displayed on the SLM for complex light field generation. Additionally, the masks are superimposed with a diffraction grating, since SLM pixels cause undesired diffraction orders, the polarisation filtering has a fidelity $< 1$, and the SLM has a fill factor $< 1$. This precaution allows the modulated light to be cleaned of the DC component. After propagation through L2, the $+1^{\text{st}}$ diffraction order is spatially separated from DC and can be filtered with an aperture (pinhole PH). The diameter is chosen that the superpixels merge into each other. After back transformation with L3, the light beam is imaged onto the MMF facet via a telescope system consisting of L4 and microscope objective MO1(Mitutoyo 80x). The incident angle is adjustable with mirror M. Proper alignment is ensured via an additional system that was presented in preliminary work [4], where the alignment procedure of the system is also explained.

On the receiver side (Bob), the emerging light field is imaged with a telescope configuration consisting of MO2 (Olympus 40x) and L5 onto a CMOS camera (IDS UI-3130). The polarisation state of the received light field is filtered according to the state on Alice's side. BS2 reunites object and reference beam in an off-axis configuration [5]. This system was used to measure TMs of up to 10 m length. We considered phase drifting effects between object and reference beam as described in the main part of this paper. For measuring the TMs of the channels of both Alice/Bob and Alice/Eve, we simply copied the receiver setup for Eve and plugged the tapped MMF piece to it.

However, when the length of an MMF is increased to for instance 100 m, phase drifts dramatically increase in frequency. The reason for this originates in fluctuations of the optical paths through both fibres (MMF and SMF). When optical paths increase in length, so does the number of target points for ambient vibrations or air flows. Fibres are particularly sensitive to such external influences and are therefore also employed as mechanical sensors [6]. In long MMF, this phenomenon can also lead to strong temporal fluctuations regarding the observed speckle field [7]. Using the components from the aforementioned optical setup with external reference, the drift cannot be measured properly anymore due to limited response times. There has already been research to compensate for the drift between object and reference paths during TM measurements on MMF. Reference-less approaches bypass the provision of a reference and do not suffer from phase drifts [8]. The stability gained is at the cost of computationally intensive optimisation algorithms that currently require a lot of time. In other work [2], phase stability is increased compared to external reference approaches, when object and reference share a common path through the MMF. Both object and reference experience the same aberrations during propagation and cancel each other when both paths interfere at the MMF output, i.e. receiver facet. In [2], it is claimed that the approach does not suffer from fundamental limitations in fibre length. However, the reference is speckled. Thus, it is not possible to measure the entire complex profile at the receiver facet, which results in 'blind spots'. Therefore, we built a common-path system in which longitudinal optical path length fluctuations between object and reference are significantly reduced. Unlike in conventional phase microscopes, the sample beam of an MMF is speckled and does not always contain a static DC component which is why Fourier phase microscopes [9] or diffraction phase microscopes [10] cannot be straightforwardly employed to an MMF. For measuring the TM of an MMF with a common-path configuration, we have built an adaptive spatial filtering using a DMD. The emerging light field on the receiver-side is split into object and reference path by BS3. The reference part needs to be spatially filtered to achieve a Gaussian shape removing the speckles. This is done by imaging the far-field onto the surface of a DMD through L6 performing a Fourier transform (denoted with $\mathcal{F}$ in Fig. S2b) The far-field is observed by a telescope consisting of L6, L7 and a CMOS camera. For each sequence of TM measurement, the region in the far-field with the highest intensity is selected and filtered by switching the respective DMD pixel to '1'. This procedure was inspired by the work from Nelsen *et al.* [11]. Afterwards, the reference beam is back-transformed through L8 ($\mathcal{F}^{-1}$) and superimposed with the object beam on the camera generating a digital off-axis hologram. In the main part of this paper, we explain how to achieve correct phase relations among the TM elements.

# 3 Channel model

The model for our considered system is based on the MIMO wiretap channel model from wireless communications [12]. An illustration of the model can be found in Fig. S3.

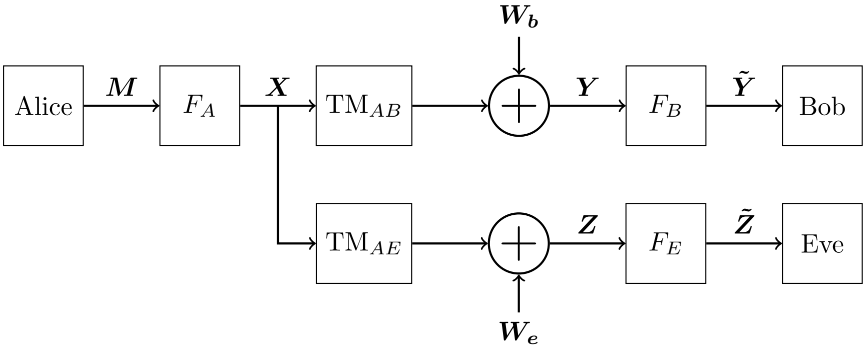


Figure S3: Considered wiretap channel model with a passive eavesdropper.

Alice wants to transmit the wiretap codeword $\tilde{\boldsymbol{M}} \in \mathbb{C}^n$ to the legitimate receiver Bob. Note that this is already the encoded and BPSK-modulated version of the actual message $\boldsymbol{M}$. She precodes the (modulated) codeword using matrix $F_A \in \mathbb{C}^{n\times n}$ into signal $\boldsymbol{X} = F_A\tilde{\boldsymbol{M}}$ that is then transmitted via the MMF. The received signals at Bob and eavesdropper Eve are given by

$$\boldsymbol{Y} = \tilde{H}\tilde{\boldsymbol{M}} + \boldsymbol{W_b} \tag{2}$$

$$\boldsymbol{Z} = \tilde{G}\tilde{\boldsymbol{M}} + \boldsymbol{W_e}\,, \tag{3}$$

respectively, where we use the shorthands $\tilde{H} = \mathrm{TM}_{AB}F_A$ and $\tilde{G} = \mathrm{TM}_{AE}F_A$. The transmission channels from Alice to Bob and Eve are represented by the matrices $\mathrm{TM}_{AB}, \mathrm{TM}_{AE} \in \mathbb{C}^{n\times n}$, respectively. In our case, this corresponds to the MMF channels, where $n$ is the number of supported modes. The terms $\boldsymbol{W_b}, \boldsymbol{W_e} \in \mathbb{C}^n$ are independent circularly symmetric complex additive white Gaussian noise (AWGN) terms with identity covariance matrix.

Next, Bob and Eve use the matrices $F_B$ and $F_E$, respectively, to process their received signals. This way, they obtain the signals $\tilde{\boldsymbol{Y}} = F_B\boldsymbol{Y}$ and $\tilde{\boldsymbol{Z}} = F_E\boldsymbol{Z}$, respectively.

The commonly used metric to analyze such channels is the *secrecy capacity*, which describes the fundamental limit of a secure communications such that Bob can reliably receive the data while simultaneously achieving an information-theoretic security against the eavesdropper [13, Chap. 3]. The secrecy capacity $C_S$ for this system is given by [13, Cor. 3.1]

$$C_S = \max_{p_{\boldsymbol{X}}} \mathbb{I}(\boldsymbol{X};\boldsymbol{Y}|\boldsymbol{Z}) = \max_{p_{\boldsymbol{X}}} \left(\mathbb{I}(\boldsymbol{X};\boldsymbol{Y}) - \mathbb{I}(\boldsymbol{X};\boldsymbol{Z})\right)\,, \tag{4}$$

where $\mathbb{I}(\boldsymbol{X};\boldsymbol{Y})$ represents the mutual information between $\boldsymbol{X}$ and $\boldsymbol{Y}$ [14].

The solution to the above problem can be found explicitly when there is an average power constraint on the input signal. In literature the common assumptions are an average power constraint over all modes, i.e., $\sum_{i=1}^{n} \mathbb{E}\left[|x_i|^2\right] \leq nP$ [15], or a per-mode average power constraint, i.e., $\mathbb{E}\left[|x_i|^2\right] \leq P$ for all $i$ [16]. However, in this work, we propose to use a different power constraint for the optical MMF system. Instead of an average power constraint, we suggest using a maximum power constraint in the form [17]

$$\sum_{i=1}^{n} |x_i(t)|^2 \leq P\,, \tag{5}$$

at all times $t$. The motivation behind this stems from the optical setup where a laser is employed at the transmitter and a camera at the receiver. First, the laser has a finite power output, and second, the camera is calibrated for a certain maximum power to avoid nonlinear fiber effects [18]. We, therefore,

get the constraint that the total emitted laser power in each time slot may not be greater than a certain power $P$, which is described in (5).

While such a peak amplitude constraint has been considered in literature before, the solution to (4), i.e., the expression of the secrecy capacity, remains an open problem for this power constraint. For the standard AWGN point-to-point channel, some characterizations of the capacity are available [19]–[21]. For the non-fading wiretap channel, there also exist initial results on the secrecy capacity achieving input distributions [22]–[24]. In particular, it is shown in [22] that the optimal input distribution has a finite support. However, the optimal input distribution is unknown for the fading wiretap channel, which we consider in this work. Therefore, we resort to a well-known BPSK transmission scheme with finite support and investigate achievable secrecy rates $R_S$ in the following section. In particular, we show that it is possible to securely transmit data at a positive rate over wiretapped MMF channels.

# 4 Secrecy Analysis – BPSK Transmission Scheme

In the following, we will provide an analysis of an achievable secrecy rate $R_S$ over wiretapped MMFs. As described in Section 3, we consider a simple transmission scheme where Alice transmits BPSK symbols with SVD precoding. Since the precoding is designed for the channel to the legitimate receiver, Bob chooses $F_B$ accordingly. On the other hand, there is a mismatch to Eve. In order to compensate this, she applies a channel inversion to the received signal, i.e., $F_E = \tilde{G}^{-1}$. By this strategy, she also diagonalises the channel with the side effect of noise amplification.

Since the resulting effective channels for both receivers are diagonal matrices, they can treat the data streams separately. For the aforementioned BPSK symbols, we can model the individual channels as binary symmetric channels (BSCs) with different bit-flip probabilities.

It should be highlighted that the calculations presented in the following are applied to the actual measured transmission matrices of the MMF channels.

## 4.1 Transmitter Model

As discussed in Section 2.2, we implement an optical precoding matrix $F_A$ as the right-singular vectors $V$ of the channel $\mathrm{TM}_{AB}$ to Bob. Combined with the corresponding reception matrix $F_B$ at Bob, the MMF channel is effectively diagonalised and separated into $n$ parallel data streams. Alice now transmits BPSK symbols on $K$ out of the $n$ parallel streams. Let $\mathcal{K} \subseteq \{1, \dots, n\}$ be an index set with $|\mathcal{K}| = K$. The indices in $\mathcal{K}$ correspond to streams that we use for data transmission.

We now consider the BPSK example which fulfills the proposed power constraint (5) from Section 3 of having a maximum power $P$ in each time slot. Specifically, we assume that the messages $\tilde{\boldsymbol{M}}$ are BPSK symbols with

$$m_i(t) \in \{-p_i, +p_i\}\,, \quad \forall i \in \mathcal{K}\,,$$

which yields

$$\sum_{i\in\mathcal{K}} |m_i(t)|^2 = P\,. \tag{6}$$

In order to transmit data, Alice selects the BPSK symbols on the individual modes (data streams) and adjusts the power of the laser accordingly. The SLM then optically applies the precoding. The resulting signal is transmitted via the MMF to Bob and Eve.

Since we do not assume knowledge of the eavesdropper's TM, Alice selects the power levels based on the singular values of the channel to Bob. Specifically, this is done by solving the optimization problem

$$\begin{aligned} \max \; & \sum_{i=1}^{K} C_i \\ \text{s.t.} \; & \sum_{i=1}^{K} p_i = P\,, \end{aligned} \tag{7}$$

where $C_i$ are the channel capacities for the individual data streams. For our considered BPSK example, these capacities will be derived in the following.

## 4.2 Receiver Model

Since we used the right-singular vectors $V$ of the channel to Bob as precoding at Alice, we need to multiply with the left-singular vectors $U$ at Bob, i.e., $F_B = U^{\mathsf{H}}$. This way, we can diagonalise the legitimate channel and separate the data streams. On the other hand, Eve's channel cannot be diagonalised this way since there is a mismatch. Thus, the eavesdropper applies a channel inversion instead, i.e., $F_E = \tilde{G}^{-1}$. With this, we get the received signals at Bob and Eve

$$\tilde{\boldsymbol{Y}} = F_B \boldsymbol{Y} = \Sigma \tilde{\boldsymbol{M}} + U^{\mathsf{H}} \boldsymbol{W_b} \tag{8}$$

$$\tilde{\boldsymbol{Z}} = \tilde{G}^{-1} \boldsymbol{Z} = \tilde{\boldsymbol{M}} + \tilde{G}^{-1} \boldsymbol{W_e} \,, \tag{9}$$

respectively.

Since $\Sigma$ is a diagonal matrix with the singular values $\sigma_i$ on the main diagonal, we effectively diagonalised the channel to Bob. Recall that the matrix $U$ with the left-singular vectors is a unitary matrix. The circular-symmetric Gaussian noise $\boldsymbol{W_b}$ therefore keeps its properties under the transformation. On the other hand, the eavesdropper will experience a noise amplification due to the channel inversion $\tilde{G}^{-1}$.

## 4.3 Secrecy Rate

In the following, we will analyze the secrecy rate of the communication system described above. Since we are using BPSK, we transform the individual mode transmissions into BSCs. The data signals $m_i$ are real and we are therefore only interested in the real part of $\hat{m}_i$. The probability of deciding for the wrong value of $m_i$ at Bob is

$$\begin{aligned} \varepsilon_{B,i} &= \Pr\left(\operatorname{Re}(\tilde{y}_i) > 0 \mid m_i = -p_i\right) && (10)\\ &= \Pr\left(\operatorname{Re}(\tilde{w}_{b,i}) > \sigma_i p_i\right) && (11)\\ &= Q\left(\frac{\sigma_i p_i}{\sqrt{\frac{1}{2}\operatorname{Re}\left(\Gamma\right)_{ii}}}\right) && (12)\\ &= Q\left(\sigma_i p_i\right)\,, && (13) \end{aligned}$$

where the simplifications stem from the facts that $\boldsymbol{W_b}$ is circularly symmetric and so is $\tilde{W_b} = U^{\mathsf{H}} \boldsymbol{W_b}$; additionally, we assume that $\boldsymbol{W_b}$ has unit variance in both real and imaginary part, i.e., its covariance matrix is $\Gamma = \mathbb{E}\left[\boldsymbol{W_b} \boldsymbol{W_b}^{\mathsf{H}}\right] = 2I$. Since $U$ is a unitary matrix, the covariance matrix of $\tilde{W_b}$ is the same.

The probability of deciding for the wrong value, based on the estimation $\hat{m}_{e,i}$ at Eve is

$$\begin{aligned} \varepsilon_{E,i} &= \Pr\left(\operatorname{Re}(\hat{m}_{e,i}) \neq m_i\right) && (14)\\ &= \Pr\left(\operatorname{Re}(\tilde{w}_{e,i}) > p_i\right) && (15)\\ &= Q\left(\frac{p_i}{\sqrt{\operatorname{Re}\left(\left(\tilde{G}^{\mathsf{H}}\tilde{G}\right)^{-1}\right)_{ii}}}\right)\,, && (16) \end{aligned}$$

where we use $\tilde{G}^{-1}\boldsymbol{W_e} \sim \mathcal{CN}\left(0, \left(\tilde{G}^{\mathsf{H}}\tilde{G}\right)^{-1}\right)$.

The capacity of a BSC with bit-flip probability $\varepsilon_i$ is given by [14, Sec. 7.1.4]

$$C_i = 1 - H_b(\varepsilon_i)\,, \tag{17}$$

where $H_b$ is the binary entropy function. The secrecy rate $R_S$ of mode $i$ is then given by the difference of the channel capacities of Bob and Eve,

$$R_{S,i} = \left[C_{\mathrm{Bob},i} - C_{\mathrm{Eve},i}\right]^{+} = \left[H_b(\varepsilon_{E,i}) - H_b(\varepsilon_{B,i})\right]^{+}\,. \tag{18}$$

Finally, the overall secrecy rate is the sum of the individual secrecy rates

$$R_S = \sum_{i\in\mathcal{K}} R_{S,i}\,. \tag{19}$$

# References


[1] R. Ryf, N. K. Fontaine, S. Wittek *et al.*, 'High-spectral-efficiency mode-multiplexed transmission over graded-index multimode fiber,' in *2018 European Conference on Optical Communication (ECOC)*, IEEE, 2018, pp. 1–3.

[2] T. Čižmár and K. Dholakia, 'Shaping the light transmission through a multimode optical fibre: Complex transformation analysis and applications in biophotonics,' *Optics Express*, vol. 19, no. 20, pp. 18 871–18 884, 2011.

[3] J. Carpenter, B. J. Eggleton and J. Schröder, '110x110 optical mode transfer matrix inversion,' *Optics Express*, vol. 22, no. 1, pp. 96–101, 2014. DOI: 10.1364/OE.22.000096.

[4] S. Rothe, H. Radner, N. Koukourakis and J. W. Czarske, 'Transmission matrix measurement of multimode optical fibers by mode-selective excitation using one spatial light modulator,' *Applied Sciences*, vol. 9, no. 1, 195, Jan. 2019. DOI: 10.3390/app9010195.

[5] E. Cuche, P. Marquet and C. Depeursinge, 'Spatial filtering for zero-order and twin-image elimination in digital off-axis holography,' *Applied optics*, vol. 39, no. 23, pp. 4070–4075, 2000.

[6] S. M. Foaleng, M. Tur, J.-C. Beugnot and L. Thévenaz, 'High spatial and spectral resolution long-range sensing using brillouin echoes,' *Journal of Lightwave Technology*, vol. 28, no. 20, pp. 2993–3003, 2010.

[7] N. Borhani, E. Kakkava, C. Moser and D. Psaltis, 'Learning to see through multimode fibers,' *Optica*, vol. 5, no. 8, pp. 960–966, 2018.

[8] M. N'Gom, T. B. Norris, E. Michielssen and R. R. Nadakuditi, 'Mode control in a multimode fiber through acquiring its transmission matrix from a reference-less optical system,' *Optics Letters*, vol. 43, no. 3, pp. 419–422, 2018.

[9] G. Popescu, L. P. Deflores, J. C. Vaughan *et al.*, 'Fourier phase microscopy for investigation of biological structures and dynamics,' *Optics letters*, vol. 29, no. 21, pp. 2503–2505, 2004.

[10] G. Popescu, T. Ikeda, R. R. Dasari and M. S. Feld, 'Diffraction phase microscopy for quantifying cell structure and dynamics,' *Optics letters*, vol. 31, no. 6, pp. 775–777, 2006.

[11] B. Nelsen, A. Kabardiadi-Virkovski, T. Baselt, C. Taudt and P. Hartmann, 'Kilohertz dynamic fourier filter for synthetic-aperture binary hologram reconstruction,' in *Practical Holography XXXIII: Displays, Materials, and Applications*, International Society for Optics and Photonics, vol. 10944, 2019, 109440U.

[12] F. Oggier and B. Hassibi, 'The secrecy capacity of the MIMO wiretap channel,' *IEEE Transactions on Information Theory*, vol. 57, no. 8, pp. 4961–4972, Aug. 2011. DOI: 10.1109/TIT.2011.2158487.

[13] M. Bloch and J. Barros, *Physical-Layer Security.* Cambridge University Press, 2011. DOI: 10.1017/CBO9780511977985.

[14] T. M. Cover and J. A. Thomas, *Elements of Information Theory*, 2nd ed. Hoboken, NJ, USA: John Wiley & Sons, Inc., 2006. DOI: 10.1002/047174882X.

[15] K. Guan, J. Cho and P. J. Winzer, 'Physical layer security in fiber-optic MIMO-SDM systems: An overview,' *Optics Communications*, vol. 408, no. June, pp. 31–41, Feb. 2018. DOI: 10.1016/j.optcom.2017.07.078.

[16] K. Guan, P. J. Winzer, A. M. Tulino and E. Soljanin, 'Physical layer security of space-division multiplexed fiber-optic communication systems in the presence of multiple eavesdroppers,' in *2015 IEEE Global Communications Conference (GLOBECOM)*, San Diego, CA, USA: IEEE, Dec. 2015. DOI: 10.1109/GLOCOM.2015.7417679.

[17] L. Li, S. M. Moser, L. Wang and M. Wigger, 'On the capacity of MIMO optical wireless channels,' *IEEE Transactions on Information Theory*, vol. 66, no. 9, pp. 5660–5682, Sep. 2020. DOI: 10.1109/tit.2020.2979716.

[18] G. P. Agrawal, *Nonlinear Fiber Optics*, 4th ed. Academic Press, 2006. DOI: 10.1016/b978-0-12-369516-1.x5000-6.

[19] A. Dytso, M. Al, H. V. Poor and S. Shamai (Shitz), 'On the capacity of the peak power constrained vector Gaussian channel: An estimation theoretic perspective,' *IEEE Transactions on Information Theory*, vol. 65, no. 6, pp. 3907–3921, Jun. 2019. DOI: 10.1109/TIT.2018.2890208. arXiv: 1804.08524 [cs.IT].

[20] A. Dytso, M. Goldenbaum, H. Poor and S. Shamai (Shitz), 'Amplitude constrained MIMO channels: Properties of optimal input distributions and bounds on the capacity,' *Entropy*, vol. 21, no. 2, 200, Feb. 2019. DOI: 10.3390/e21020200.

[21] A. Favano, M. Ferrari, M. Magarini and L. Barletta, 'The capacity of the amplitude-constrained vector Gaussian channel,' in *2021 IEEE International Symposium on Information Theory (ISIT)*, IEEE, Jul. 2021, pp. 426–431. DOI: 10.1109/ISIT45174.2021.9518071. arXiv: 2101.08643 [cs.IT].

[22] O. Ozel, E. Ekrem and S. Ulukus, 'Gaussian wiretap channel with amplitude and variance constraints,' *IEEE Transactions on Information Theory*, vol. 61, no. 10, pp. 5553–5563, Oct. 2015. DOI: 10.1109/TIT.2015.2459705.

[23] L. Barletta and A. Dytso, 'Scalar Gaussian wiretap channel: Properties of the support size of the secrecy-capacity-achieving distribution,' in *2021 IEEE Information Theory Workshop (ITW)*, IEEE, Oct. 2021. arXiv: 2109.01566 [cs.IT].

[24] A. Favano, L. Barletta and A. Dytso, *On the capacity achieving input of amplitude constrained vector Gaussian wiretap channel*, Feb. 2022. arXiv: 2202.00586 [cs.IT].
